# Data on the optimized sulphate electrolyte zinc rich coating produced through in-situ variation of process parameters

**DOI:** 10.1016/j.dib.2017.10.010

**Published:** 2017-10-06

**Authors:** Ojo Sunday Isaac Fayomi

**Affiliations:** a Department of Mechanical Engineering, Covenant University, P.M.B. 1023, Ota, Nigeria; b Department of Chemical, Metallurgical and Materials Engineering, Tshwane University of Technology, P.M.B. X680, Pretoria, South Africa

**Keywords:** Coating thickness, Weight gained, Sulphate electrolyte, Voltage, Microhardness

## Abstract

In this study, a comprehensive effect of particle loading and optimised process parameter on the developed zinc electrolyte was presented. The depositions were performed between 10–30 min at a stirring rate of 200 rpm at room temperature of 30 °C. The effect of coating difference on the properties and interfacial surface was acquired, at a voltage interval between 0.6 and 1.0 V for the coating duration. The framework of bath condition as it influences the coating thickness was put into consideration. Hence, the electrodeposition data for coating thickness, and coating per unit area at constant distance between the anode and cathode with depth of immersion were acquired. The weight gained under varying coating parameter were acquired and could be used for designing and given typical direction to multifunctional performance of developed multifacetal coatings in surface engineering application.

**Specification Table**

**Table:** 

Subject area	*Materials Engineering*
More specific subject area	*Surface Science and Engineering*
Type of data	*Table, image*
How data was acquired	The deposition took place in a constructed electrodeposition sequence cell containing five steps according to the principle of electrolytic co-deposition route from pre treatment to post treatment. The coating thickness, weight gained, coating per unit area were measured using coating thickness gauge and weighing balance for the weight gain. The coating per unit area was obtained from the calculated value of the coating thickness for each value of deposited matrix.
Data format	Raw, Analyzed
Experimental factors	The particles were measured appropriately and electrolyte pH was obtained before the deposition was done and required data acquired.
Experimental features	The depositions were performed between 10–30 min at a stirring rate of 200 rpm at room temperature of 30 °C. The effect of coating difference on the properties and interfacial surface was acquired, at a voltage interval between 0.6 and 1.0 V for the coating duration. The framework of bath condition as it influences the coating thickness was put into consideration.
Data source location	Department of Chemical, Metallurgical and Materials Engineering, Tshwane University of Technology, Pretoria, South Africa and Mechanical Engineering, Covenant University, Ota Ogun State, Nigeria
Data accessibility	Data are available within this article

**Value of the data**

• The given data will show author in the field of surface science the correlation and effect between the zinc electrolyte and the continuous metal matrix induced electrolyte in a given engineering component.
• The data obtained for the zinc electrolyte can be used as inference to determine the anomalous metal matrix co-deposition coating for other intended nano-particle coating.
• The data can be used to examine the relationship between the process variable for instance (voltage and time) as it affect the nature of coating properties produced.
• The data could be used at investigating the coating progression between the coating thickness, weight gain and the surface area of adsorbed deposits
• The data obtained can be used in investigating the strengthening behaviour of particulate in an electrolyte relating to its mechanical characteristics.

## 1 Data

The coating thickness, weight gained, coating per unit area at constant distance between the anode and cathode with depth of immersion were collected and a unique set of experimental frame work data were generated. The depositions process was performed between 10 and 30 min at a stirring rate of 200 rpm at ambient temperature of 30 °C. The data acquired from spectrometer analysis of the mild steel is presented in Table 1. The coating depositions was run twice on two separate mild steel substrate from single electrolyte for all set of sample matrix to ascertain its deposition. The variable coating thickness, weight gained, coating per unit area were each acquire twice and the average taken as representative data for better precision. Also, data showing deposited variable in term of voltage and time of deposition was gathered (see Table 2, Table 3, Table 4, Table 5).

**Table 1:** Data showing the composition of steel substrate used.

**Element**	**%Content**	**Element**	**%Content**	**Element**	**%Content**
C	0.134	Mo	0.083	Ti	< 0.002
Si	0.119	Ni	0.019	V	0.0048
Mn	0.237	Cu	0.044	W	0.024
P	< 0.003	Al	0.050	B	> 0.016
S	> 0.156	Co	0.012	Sn	0.0046
Cr	0.094	Nb	< 0.005	Fe	97.70

**Table 2:** Experimental data showing electrodeposition parameters and results for zinc plated mild steel.

**Sample Numbers**	**Time (min)**	**Coating Thickness (μm)**	**Weight Gained (g)**	**Coating per unit area (mg/mm**^**2**^**)**	**Voltage (V)**
Zn 1	10	0.95	0.041	0.017	0.6
Zn 2	15	2.23	0.123	0.045	0.6
Zn 3	20	3.09	0.142	0.056	0.6
Zn 4	25	5.54	0.273	0.104	0.6
Zn 5	30	5.72	0.291	0.109	0.6
Zn 6	10	1.58	0.069	0.028	0.7
Zn 7	15	2.50	0.109	0.044	0.7
Zn 8	20	3.51	0.165	0.064	0.7
Zn 9	25	4.26	0.189	0.076	0.7
Zn 10	30	4.28	0.220	0.082	0.7
Zn 11	10	1.97	0.088	0.035	0.8
Zn 12	15	3.72	0.164	0.066	0.8
Zn 13	20	3.80	0.181	0.070	0.8
Zn 14	25	5.76	0.295	0.110	0.8
Zn 15	30	7.34	0.374	0.140	0.8
Zn 16	10	6.40	0.299	0.117	0.9
Zn 17	15	7.67	0.344	0.136	0.9
Zn 18	20	8.20	0.359	0.145	0.9
Zn 19	25	17.1	0.797	0.302	0.9
Zn 20	30	18.7	0.825	0.314	0.9
Zn 21	10	6.50	0.310	0.128	1.0
Zn 22	15	7.69	0.355	0.152	1.0
Zn 23	20	8.40	0.397	0.164	1.0
Zn 24	25	12.4	0.799	0.324	1.0
Zn 25	30	19.2	0.840	0.341	1.0

**Table 3:** Experimental data showing summarized data of plated samples for constant plating time at various voltages.

**Sample No**	**Deposition Time (min)**	**Deposition Voltage (V)**	**Plating Effects**	**Weight of Deposition (g)**	**Thickness of Deposition (μm)**
Zn 1	20	0.6	Diffused reflection	0.142	3.09
Zn 2	20	0.7	Diffused reflection	0.165	3.51
Zn 3	20	0.8	Fairly bright reflection	0.181	3.80
Zn 4	20	0.9	Bright reflection	0.359	8.20
Zn 5	20	1.0	Bright reflection	0.395	8.40
Zn (As-received)	20	–	–	–	–

**Table 4:** Experimental data showing electrodeposition parameters and results for Zn-Al plated mild steel.

**Sample Numbers**	**Time (min)**	**Coating Thickness (μm)**	**Weight Gain (g)**	**Coating per unit area (mg/mm**^**2**^**)**	**Voltage (V)**
Zn-Al 1	10	0.970	0.043	0.018	0.6
Zn-Al 2	15	2.260	0.126	0.048	0.6
Zn-Al 3	20	3.120	0.144	0.060	0.6
Zn-Al 4	25	5.520	0.270	0.102	0.6
Zn-Al 5	30	5.700	0.289	0.106	0.6
Zn-Al 6	10	1.600	0.073	0.030	0.7
Zn-Al 7	15	2.530	0.111	0.047	0.7
Zn-Al 8	20	3.520	0.166	0.065	0.7
Zn-Al 9	25	4.300	0.193	0.080	0.7
Zn-Al 10	30	4.300	0.223	0.085	0.7
Zn-Al 11	10	1.950	0.086	0.032	0.8
Zn-Al 12	15	3.740	0.166	0.068	0.8
Zn-Al 13	20	3.820	0.183	0.072	0.8
Zn-Al 14	25	5.740	0.293	0.108	0.8
Zn-Al 15	30	7.360	0.376	0.142	0.8
Zn-Al 16	10	6.430	0.302	0.120	0.9
Zn-Al 17	15	7.700	0.348	0.139	0.9
Zn-Al 18	20	8.230	0.362	0.148	0.9
Zn-Al 19	25	17.05	0.792	0.297	0.9
Zn-Al 20	30	18.73	0.828	0.318	0.9
Zn-Al 21	10	6.530	0.313	0.131	1.0
Zn-Al 22	15	7.710	0.357	0.154	1.0
Zn-Al 23	20	8.430	0.400	0.167	1.0
Zn-Al 24	25	12.45	0.804	0.328	1.0
Zn-Al 25	30	19.28	0.848	0.349	1.0

**Table 5:** Experimental data showing summarized data of Zn-Al plated samples for constant plating time at various voltages.

**Sample No**	**Deposition Time (min)**	**Deposition Voltage (V)**	**Plating Effects**	**Weight of Deposition (g)**	**Thickness of Deposition (μm)**
Zn-Al 1	20	0.6	Diffused reflection	0.144	3.12
Zn-Al 2	20	0.7	Diffused reflection	0.166	3.52
Zn-Al 3	20	0.8	Diffused reflection	0.183	3.82
Zn-Al 4	20	0.9	Bright reflection	0.362	8.32
Zn-Al 5	20	1.0	Bright reflection	0.400	8.43
Zn (As-received)	20	–	–	–	–

## 2 Experimental design, materials and methods

**Fig. 1:**
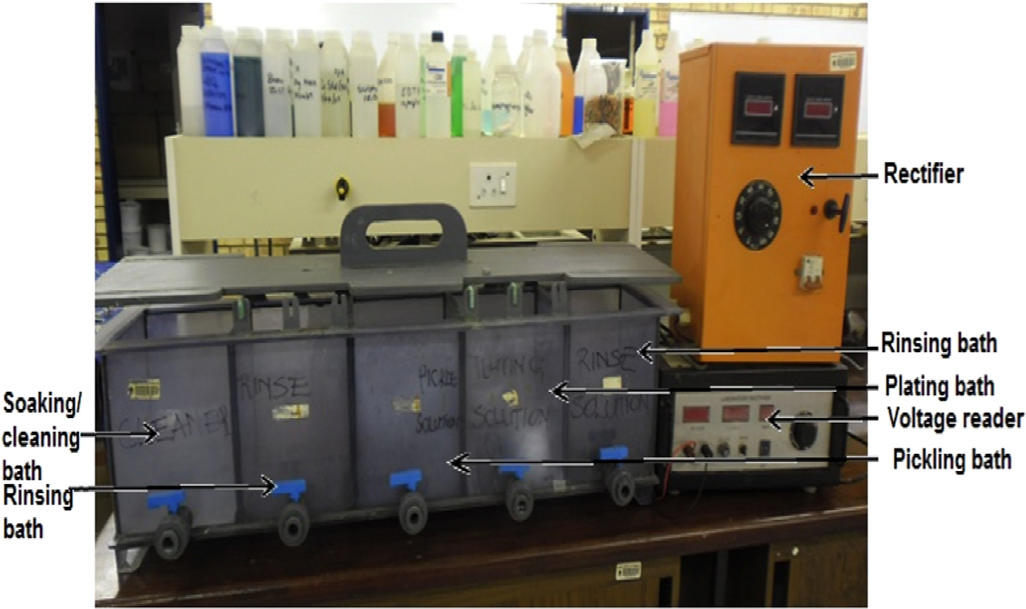
Electroplating setup.

**Fig. 2:**
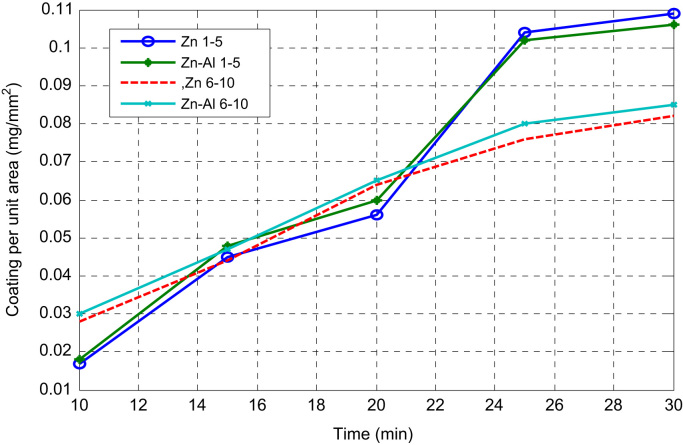
Experimental trend of variation in coating per unit area with time of deposition sample 1–10.

**Fig. 3:**
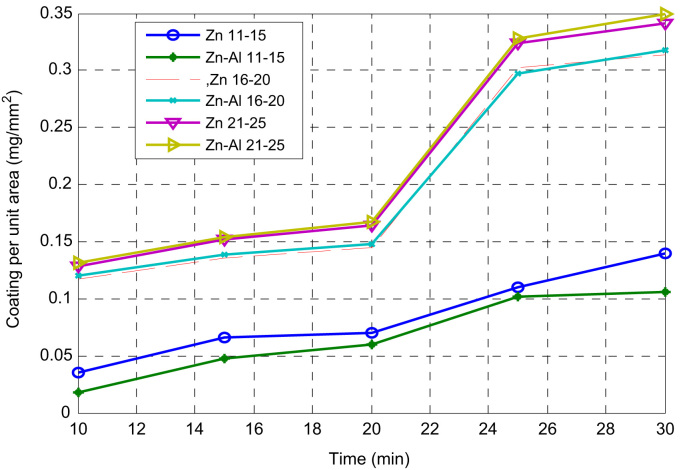
Experimental trend of variation in coating per unit area with time of deposition sample 11–25.

An electrocodeposition system used for this set up is shown in Fig. 1. The dimension of the mild steel (substrate) used was 45 mm × 40 mm × 20 mm. Zinc sheets of 85 mm × 45 mm × 5 mm with 99.99% were prepared as anodes as described [1]. The mild steel specimens were polished mechanically, degreased and rinsed with water as described [2], [3]. Powder purchased from Sigma Aldrich was used as received. The bath formulations were prepared a day before and stir continuously at the rate of 200 rpm to obtain homogeneous solution. The bath compositions used for the different coating matrix is as follows 120 g/L of ZnSO_4_, 30 g/L of K_2_SO_4_, 20 g/L of Al_2_O_3_ 15 g/L of 0.5 g/L of 2- Butyne 1,4 diol, 0.2 g/L of and 5 g/L of Thiourea. H_3_BO_4_. The choice of the deposition parameter is in line with the study from previous work of some authors [4], [5]. The dispersion strengthening behaviour which often causes change in coating performance [6] helps to obtained coating thickness, weight gained, coating per unit area generated and presented in Fig. 2, Fig. 3.
