# Supplementary material for: Data on the optimized sulphate electrolyte zinc rich coating produced through in-situ variation of process parameters
Source: Data Brief. 2017 Oct 6;16:141–6. doi: 10.1016/j.dib.2017.10.010 (PMC5699877; doi:10.1016/j.dib.2017.10.010)
Supplement: Supplementary file 1 — Supplementary material [file mmc1.docx]

***COVER LETTER/CONFLICT OF INTEREST ATTESTATION***

*11th September, 2017*

*The Editor-in-Chief*

*Data in Brief*

***Subject:***  ***NO CONFLICT OF INTEREST***

*Dear Sir,*

*This serve to notify you that the manuscript is original and there is no conflict of interest of any kind regarding the manuscript Data on the optimized sulphate electrolyte zinc rich coating produced through in-situ variation of process parameters*

*Sincerely yours,*

*Dr. OSI Fayomi*

*Department of Chemical, Metallurgical and Materials Engineering
Tshwane University of Technology
P.O. Box X680*

*Pretoria
South Africa*
